# Exploring environmental measures in disability: Using Google Earth and Street View to conduct remote assessments of access and participation in urban and rural communities

**DOI:** 10.3389/fresc.2022.879193

**Published:** 2022-08-05

**Authors:** Tom Seekins, Meg A. Traci, Emily C. Hicks

**Affiliations:** Rural Institute for Inclusive Communities, University of Montana, Missoula, MT, United States

**Keywords:** accessibility, participation, environment, rural penalty, behavioral ecology, disability

## Abstract

The Americans with Disabilities Act has been in place since 1990. Yet, we still do not know the actual levels of accessibility in the nation, how access varies across communities or over time, or how it influences participation in community life. The present two studies explored the use of Google Earth (GE) and Google Street View (GSV) imagery as a database for examining the accessibility of rural and urban cities and towns in the United States. We developed procedures for selecting places in a community to observe multiple access features. Study 1 reports the findings from assessments of 25 communities across 17 states. We observed ≈50,000 m (31 miles) of pathways through the observed places. The Combined Access Score (CAS) averaged 65% across these communities. In Study 2, we evaluated 22 towns and cities in a large rural state. We observed ≈77,000 m (48 miles) of pathways through the Central Business Districts observed as core areas connecting people to community life. The CAS averaged 83.9% across these communities. We noted a Rural Access Penalty (RAP), such that rural areas tended to be less accessible, leading to less community participation. The method for using GSV to examine accessibility is discussed. This study demonstrates an inexpensive and reliable method for evaluating the accessibility of communities and participation in them. Future research should be conducted to gather a larger sample of communities in order to create a baseline from which to monitor changes in accessibility of infrastructure over time.

## Introduction

The design and organization of a community's environment can significantly influence the degree to which people that experience mobility limitations associated with chronic conditions have opportunities to participate in community life (1–5). In the United States, several laws, policies, and programs focus on arranging the environment to increase participation. For example, the Americans with Disabilities Act (ADA) sets rules for employment, communications technology, and the built environment intended to promote participation in community life. Governments and private entities have invested significant resources to build public infrastructure that approaches universal access in rural and urban areas (6). Still, we know surprisingly little about the extent to which these efforts have succeeded or how they function across the 30,000 urban and rural communities in the United States.

Several scientists have demonstrated methods for measuring environmental factors affecting participation. For example, Carlsson and colleagues (7) developed a housing usability screening instrument that identifies housing accessibility problems. Whiteneck and colleagues (8) developed a self-report questionnaire (the CHIEF) to assess the frequency and magnitude of barriers that keep people from engaging in desired activities. Gray and colleagues (1, 9) have demonstrated a self-report protocol for recording the accessibility of environments people visit. Nary et al. (10) used direct observation to evaluate the accessibility of several fitness facilities. Others have used the Behavioral Risk Factor Surveillance System to assess the visitability of homes [e.g., 15] and have estimated the likelihood of a house being occupied by a person with a significant impairment during the lifetime of the house (11). Finally, Seekins et al. (12, 13) used data collected by direct observation of businesses randomly selected from those in a small city and all towns between 2,500 and 10,000 residents in Montana. Still, few of these have been taken to scale; perhaps, at least in part, because the methods would require significant resources and organization to collect data across distant sites.

Recently, several researchers have used Google Street View (GSV) to assess various characteristics of public spaces. For example, Ben-Joseph and colleagues (14) used GSV as a publicly available database to assess environmental conditions promoting health. Similarly, Rundle and colleagues (15) used GSV to assess characteristics of neighborhood vitality (e.g., aesthetics, physical disorder, infrastructure for travel, sidewalk amenities, and social and commercial activity). GSV has been used to examine disaster preparedness (16), pedestrian injury (17), and supportiveness of physical activity (18). In a recent review of over 600 papers that used street level imagery, Biljecki and Ito (19) found that most studies used GSV to describe the built environment for a range of purposes and concluded that street level imagery is “…now clearly an entrenched component of urban analytics and GIScience” (p. 1).

We report two studies aimed at developing methods and procedures for using GE and GSV, large geospatial digital data bases representing images of the physical structures on or near roadways in the United States, to assess the accessibility of communities. Study 1 reports the findings of assessments of 25 towns and cities in 17 states and the District of Columbia. Study 2 reports the findings from assessments of 22 towns and cities in one, large rural state developed within a state program of technical assistance to community action teams (CATs) working on community development plans.

## Study 1

Researchers routinely monitor features of natural ecologies to assess the health of places and their populations (20). Cities and towns are ecological habitats for human populations. The accessibility of our cities and towns can affect the health and participation of people with disability. The aim of Study 1 was to explore procedures for conducting remote monitoring of the accessibility of communities of various sizes located at distance from the observer (21, 22). Such a monitoring system requires procedures for both selecting places to observe within a community and procedures for observing the features of those places. We chose GE and GSV as the source of data from which to extract observation to assess the accessibility of cities and towns.

### Sample

We chose a convenience sample of 25 towns and cities in 17 states and the District of Columbia to evaluate. These were chosen in four groups; including (1) seven that were among the towns Seekins and colleagues (13) assessed; (2) 16 were hometowns of elected and appointed officials with jurisdiction over the Americans with Disabilities Act), (3) and two were chosen at convenience to explore more diverse communities. The cities and towns observed had populations ranging from 235 to 2.59 million (*mean* = 181,030.60, *SD* = 521,819.36). The locations observed in each town included access features of the sidewalks along ≈2,000 m around the city hall and the building entrance along 500 m of the selected sidewalks.

### Procedures

#### Places observed

Seekins et al. (12, 13) assessed the accessibility of communities by directly observing the accessibility of businesses selected randomly from among all businesses in a community. In this study, we chose to assess the accessibility of a central area of incorporated cities and towns. While incorporated places vary in many ways, each has a city hall or comparable administrative office. We reasoned that the area around the city hall should be among the most accessible areas in any city or town. That area is often the civic center of a city or town around which commercial activities take place. The left panel of Supplementary Image 1 shows a Google Earth satellite view of such an area of one town.

The concept of accessibility includes elements of the built environment that support or hinder the participation of those with mobility impairments. Accessibility can be viewed from a legal or a functional perspective. A legal perspective specifies exact criteria to use in determining whether an arrangement complies with a law (e.g., 32″ doorways). A functional perspective assesses the “usability” of an environmental arrangement (e.g., 28). As implied, usability suggests a wider range of acceptable arrangements that still allow a person to achieve the aim, albeit with more effort (e.g., a ramp with a gentle slope vs. 1:12 slope ratio). Usability is more of a judgment of accessibility than a precise measurement of legal requirements. This study focused on assessing the usability of physical elements of the environment observable from images presented in GSV. In this work, we use the terms accessibility and usability interchangeably.

We used GSV to assess the usability/accessibility of sidewalk pathways and business entries. Brooke (23) suggests using a five-point Likert-type scale for assessing usability of any product. We derived an accessibility rating instrument for assessing the usability of the physical environment of cities and towns based on one developed by Seekins et al. (12, 13). Observers rated the usability/accessibility of curb cuts (CC) and sidewalk segments (SS), and entry ways (EW) and doorways (DW) of non-residential buildings using a five-point, anchored rating scale. The anchors included ratings of “0” or access failure, “1” for access risk, “2” for obstructed, “3” for poorly maintained, and “4” for a clear and accessible pathway. Each anchor included specific definitions for each feature with examples. If an image lacked focus sufficient to see a feature clearly, its accessibility was not rated, and a null symbol was recorded.^1^

An observer applied the scales to record observations of pathway usability along sidewalks on each side of the 1,000-m pathway for a total of about 2,000 m per place. They applied the scales to the buildings along the 250-m pathway for a total of about 500 m per place. They also tallied the number of people present as pedestrians, the number who used personal mobility devices (e.g., wheelchair, scooter, cane, guide animal, etc.), and the number using other wheeled devices along the 500-m pathway. We also noted features of access and public participation. Features included temporary obstacles, such as safety cones blocking the sidewalk, and permanent barriers, such as lamp posts blocking the sidewalk (see right panel of Supplementary Image 1). Finally, we collected pictures of unique arrangements, situations, and features.

As this exploratory research unfolded, we noted both new features that could be observed and new situations for scoring. When we adopted significant new measures or procedures, we rescored previously observed places.

#### Observational protocol

An observer secured the address of the city hall of each place, along with data on the population of the community to be observed from its official website. Then the observer opened the Google Earth program on a computer and entered the address into the search box. Once the city hall or equivalent place was located, the observer used GSV to mark the location of the nearest street intersection with the thumbtack tool. Next, the observer left GSV and oriented to the layout of the city using the Google Earth's satellite view. Beginning at the position previously marked, the observer used the pathway tool to draw a line of ≈1,000 m of roadway for observing the accessibility of the sidewalk system. The line was drawn from the target address down the center of the street leading toward the area of greatest development and looped back through the area to where it began, when possible. If a city boundary or natural end was encountered, or if there was limited street view availability, the line was extended in the direction of the next most developed area or until a total of 1,000 m was reached. A second line of 250 m was traced from the same starting point along the same pathway as the sidewalk segment for rating the accessibility of building entries and doorways, and for recording the people present. Finally, an image of the city with the path drawn was saved for reference.

Next, the observer navigated through the visual images presented by GSV on the computer screen, moving along the 1,000-m line rating the accessibility of each curb ramp (CC) and sidewalk segment (SS) on one side of the line. Upon returning to the beginning of the path, the observer followed the path again on the other side. Every curb ramp passed along the path of travel was rated. At each corner, each ramp or corner adjacent to the street was rated, progressing clockwise from the straight-ahead path of travel. Once the curb ramps were rated, the observer rated the accessibility of the segment of sidewalk on the line to the next intersecting street, alley, or other vehicular roadway. As such, observers rated ≈2,000 m (1.24 miles) of the sidewalk system, and the buildings and people along 500 m of those same sidewalks in each city or town. A complete observation—from preparing the observation files through rating a town's accessibility and participation, to saving and accounting for the data—took ≈2 h for each city.

#### Inter-observer reliability

Seekins et al. (12) reported inter-observer agreement that averaged 91% using the original, direct-observation protocol and measures. Seekinset al. (24) reported inter-observer agreement that averaged 84% across all GSV ratings of usability/accessibility, including 96% for curb cut, 89% for sidewalk segment, 80% for doors, and 50% for entryway ratings. Agreement on people observed was 93%. Correspondence between observations made using GSV and those made directly averaged 85%.

#### Data analysis

While this was an exploratory project, we were guided by a hypothesis that the population of a community (e.g., rural, or non-metropolitan status) would statistically predict accessibility, and that the accessibility of a community would predict participation by individuals using mobility devices. As data analysis proceeded, we recognized the possibility that several metrics, derived from the primary data might also be related to accessibility, participation, or to newly derived measures. These are described below.

The ratings of the usability/access features for a town or city were entered into an Excel spreadsheet. Ratings were converted to percentage scores for CC and SS along streets, and for doorways and entries of non-residential buildings. The scores for CC and SS combined into an average Pathway Access Score. The ratings for entries and doorways were combined into an average Building Access Score. Pathway and Building Access Scores were multiplied to create a Combined Access Score. These scores were calculated for each community and for all communities combined.

Similarly, we tallied the number of temporary and permanent obstacles observed in each community. We also tallied the number of people observed, the number of people using mobility devices, and the number of people pushing or pulling other wheeled devices. Again, these scores were calculated for each community and for all communities combined.

#### Derived variables

We also derived new measures from the data that led to additional hypotheses that we examined (see Table 1 for variable names and definitions). For example, we reasoned that the fewer the number of interruptions in any pathway, the more likely people with mobility devices would be present. Therefore, we explored three measures of such interruptions. First, we derived Access Risk and Access Failure Indices. Access Risk was scored as a “1” whenever a feature presented a potential danger of falling or getting stuck due to poor conditions of the feature (e.g., cracked sidewalk) or forcing a person to leave the sidewalk and enter the street in order to circumvent a permanent or temporary obstacle. Access Failure was scored as “0” whenever there was a barrier (e.g., telephone pole in pathway or no curb ramp) that would block a person using a mobility device from continuing along a Path of Travel (i.e., inaccessible) with no visible options. The Risk Index was derived by counting the number of ratings of “1” in either sidewalk or curb cut ratings observed per 1,000 m. The Access Failure Index was derived by counting the number of ratings of “0” in either sidewalk or curb cut ratings per 1,000 m.

**Table 1:** Definitions of selected terms and concepts.

**Terms used**	**Definition**
Public Participation	Presence of an individual in an open public space
Rule of Proportional Participation	The ideas that the proportion of people who use mobility devices present at any time in a given place ought to be proportionate to their prevalence in the population as a whole; environmental factors should explain deviations from this proportion
Pathway of Travel	A line between two points that a person might follow to get from one end to the other
Sidewalk Segment	That portion of a pathway from the edge of an intersecting motor way to the next intersecting motorway
Curb (Cut) Connector	A short ramp cutting through a curb or built up to it
Building Entryway	Any access point to a building or portion of a building or facility used for the purpose of entering. An entrance includes the approach walk, the vertical access leading to the entrance platform, the entrance platform itself, and vestibule if provided, the entry door or gate, and the hardware of the entry door or gate
Doorway	The entry door or gate, and the hardware of the entry door or gate
Pathway Access Score	The percentage of combined sidewalk and curb ramp ratings
Building Access Score	The percentage of combined entry and doorway ratings
Index of Building Access	The proportion of buildings which the entry and the doorway each receive an Access Rating of at least “1”
Combined Access Score	The percentage of the total possible points of all access ratings, including curb cuts, sidewalk segments, building entries, and building doorways
Temporary Obstacle	Obstructions to the path of travel that could be moved, such as a utility truck parked on a sidewalk to repair an overhead wire
Right of Way Obstruction	The permanent installation of a fixed object (e.g., fire hydrant) in a curb cut or sidewalk so that it blocks the passage along a path of travel
Permanent Barrier	A barrier in the path of travel that cannot be moved without significant effort, such as a utility pole placed in a curb cut
Access Risk	A feature of the pathway puts an individual at risk (e.g., forces one into traffic) to navigate a barrier and continue on the pathway
Access Failure	A barrier blocks progress along a Pathway of Travel (i.e., inaccessible)
Threat Access Ratio	The inverse of the proportion of Access Risks and Failures to the total opportunities for passage.
Access Island	Areas where there is good pathway and building access, but it comes to an abrupt end
Access Barren	An area in which both Pathway and Building Access Scores fall below 40%
Access Desert	Areas in which the Pathway Access Score exceeds 80% but the Building Access Scores fall below 40%
Rural Access Penalty	The discrepancy in accessibility found between urban and rural areas; cities above and below 50,000 population; and then above and below 10,000

Second, we reasoned that the experience of accessibility might be influenced by the proportion of risks and failures to the opportunities encountered in a given pathway rather than their simple frequency. We derived a Threat Access Ratio (TAR) by taking the inverse of the sum of the number of access risks and failures encountered as a proportion of opportunities (i.e., number of curb cut and sidewalk ratings). Similarly, we derived an Available Building Ratio by calculating the inverse of the number of buildings that were rated inaccessible to the total number of buildings.

Next, we plotted the scores for each measure across communities, rank-ordered by population. We examined the relationships between population and access using regression and Mann-Whitney *U* test, and we used Kendall's *t* to examine differences in access and participation. Alpha was set at 0.05.

#### The rule of proportional participation

In the process of examining the relationship between accessibility of a place and the presence of people who use mobility devices, we recognized the need for a standard metric to compare communities with varying populations. We developed the Rule of Proportional Participation (RPP), the idea that the proportion of people who use mobility devices present at any time in a given place ought to be proportionate to their prevalence in the population as a whole; environmental factors should explain deviations from this proportion. LaPlante and Kaye (25) report that rate as 4.5% of non-institutionalized individuals 6 years old and older. Table 1 lists and defines the RPP, as well as other terms for measures and outcomes reported here.

### Results

Overall, we evaluated 25 towns and cities in 17 states and the District of Columbia. We observed ≈50 km (31 miles) of pathways through the observed places, including: 1,100 curb ramps, 513 sidewalk segments, and 233 buildings (with 225 entries and 194 doorways that were rated). The Combined Access Score averaged 65% across these communities, including an average Pathway Access Score of 67% and an average Building Access Score of 63%. Only one community received a perfect score across these categories.

#### Access and population

**Figure 1:**
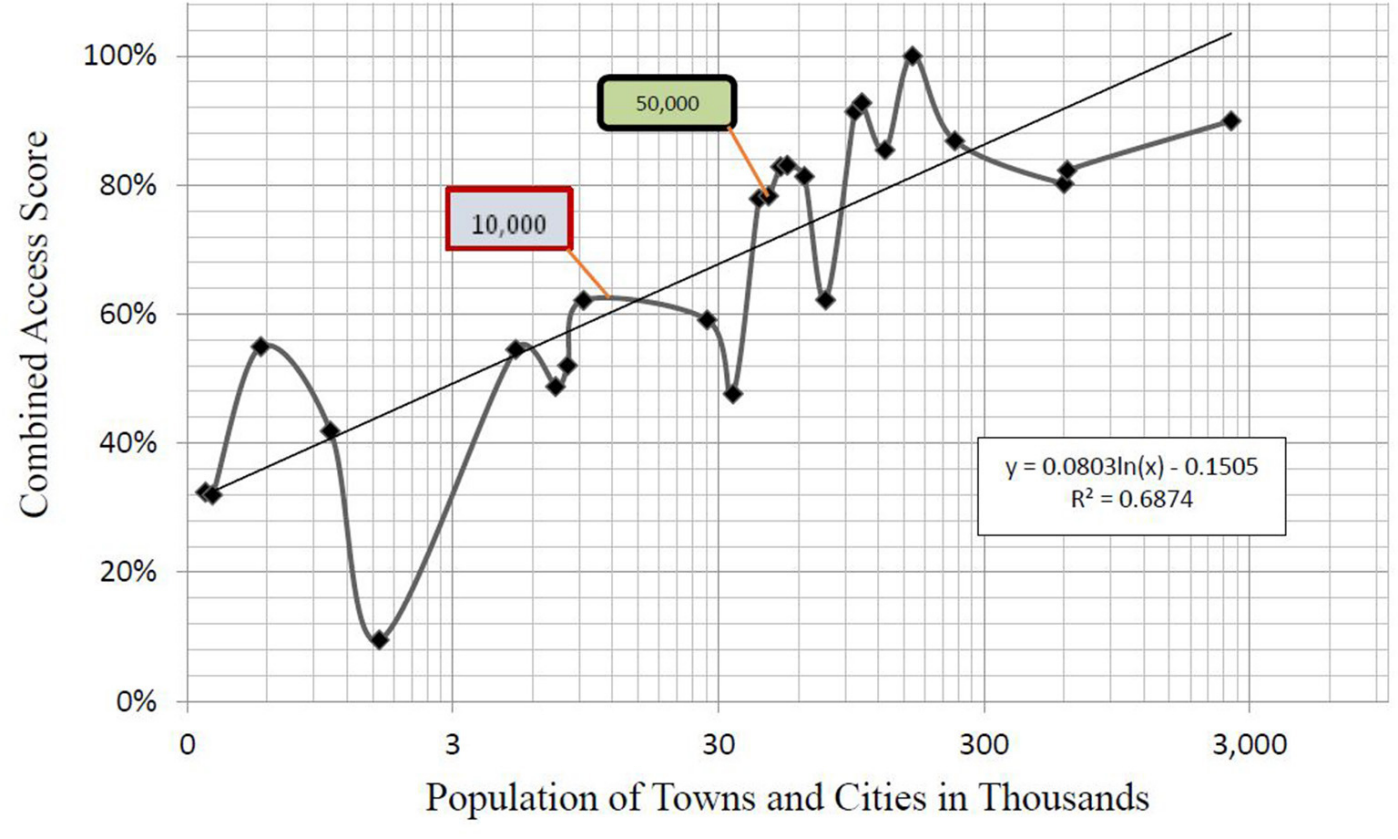
Access and Population—This figure displays the combined access scores of 25 cities and towns by the log of their populations. Populations account for over 60% of the variance in access. The points at which populations exceed 10,000 and 50,000 are marked.

Because the population of the towns and cities varied by over 2.5 million, we assessed the relationship between the population of a community and the Combined Access Score (CAS) by plotting it on a logarithmic scale. Figure 1 portrays the data on a logarithmic scale for population (*R*^2^ = 0.69). One box marks the point between places with a population below and above 10,000 (between non-metropolitan, non-core, and core-based counties) and another box indicates the point above the 50,000-population threshold (metropolitan counties). The towns under 50,000 averaged an access score of 42% while the cities above the 50,000-threshold averaged 76% [U(N_MetropolitanCAS_ = 18, N_Non−MetropolitanCAS_ = 7) = 17, *z* = 2.83, *p* < 0.05]. No town with a population <50,000 exceeded a CAS of 78%. All except one city with a population >50,000 exceeded a CAS of 80%.

#### Access features

**Figure 2:**
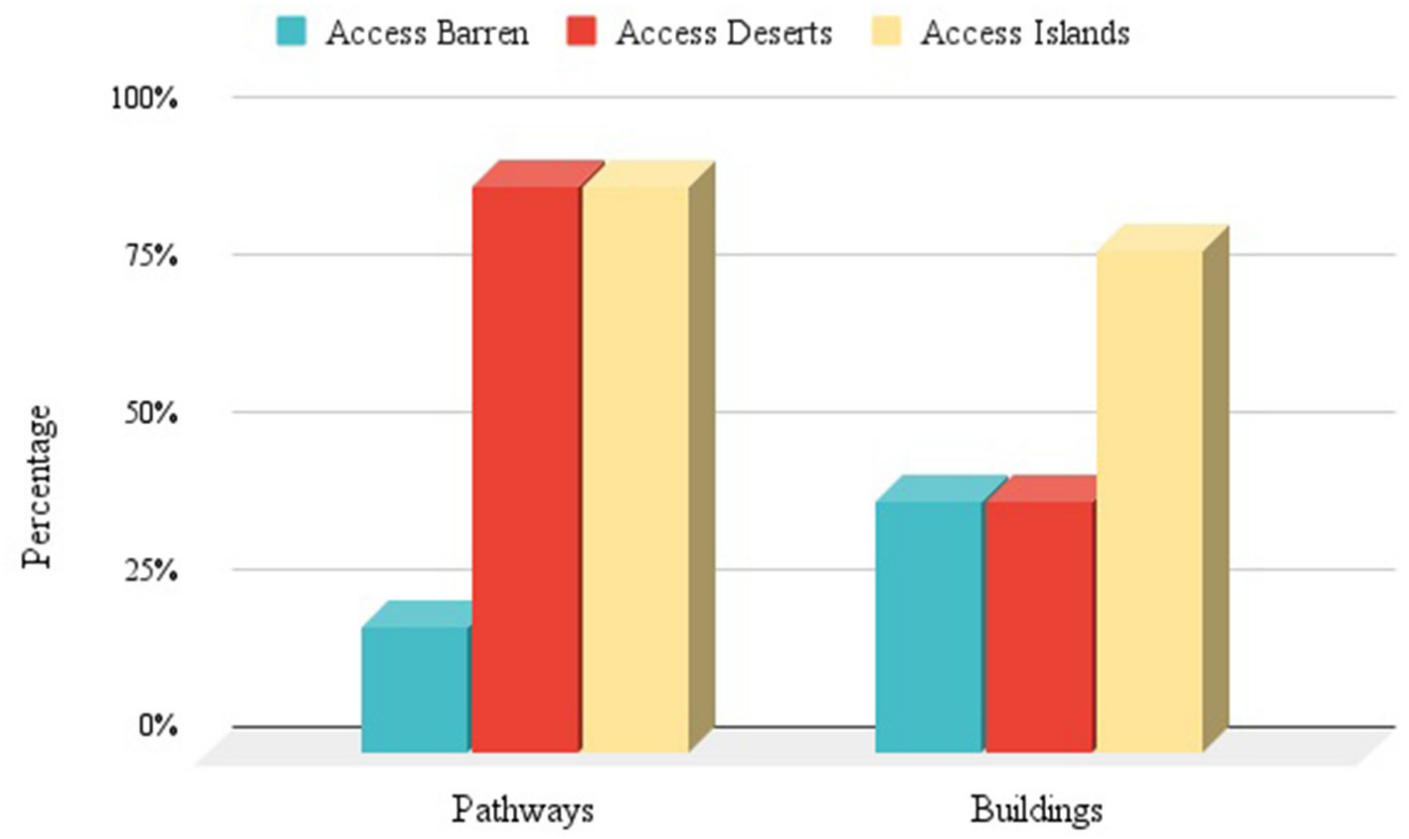
Three Derived Concepts—shows three concepts derived from the data, including Access Islands (yellow) in which pathway and building access are both high; Access Deserts (red) in which pathway access is high but building access is low; and Access Barrens (blue) in which both pathway and building access are low. In Access Islands, a person using a mobility device can move around most or all of an area and get into most or all buildings. In Access Deserts, one can move around most of an area but cannot get into many of the buildings. In Access Barren, it is difficult to move around an area or get into many buildings.

We noted three new categories for classifying access features in a community. Figure 2 shows Access Islands (areas with highly accessible pathways and buildings), Access Deserts (highly accessible pathways but fewer than 40% of buildings are accessible), and Access Barrens (low pathway accessibility and low building accessibility).

**Figure 3:**
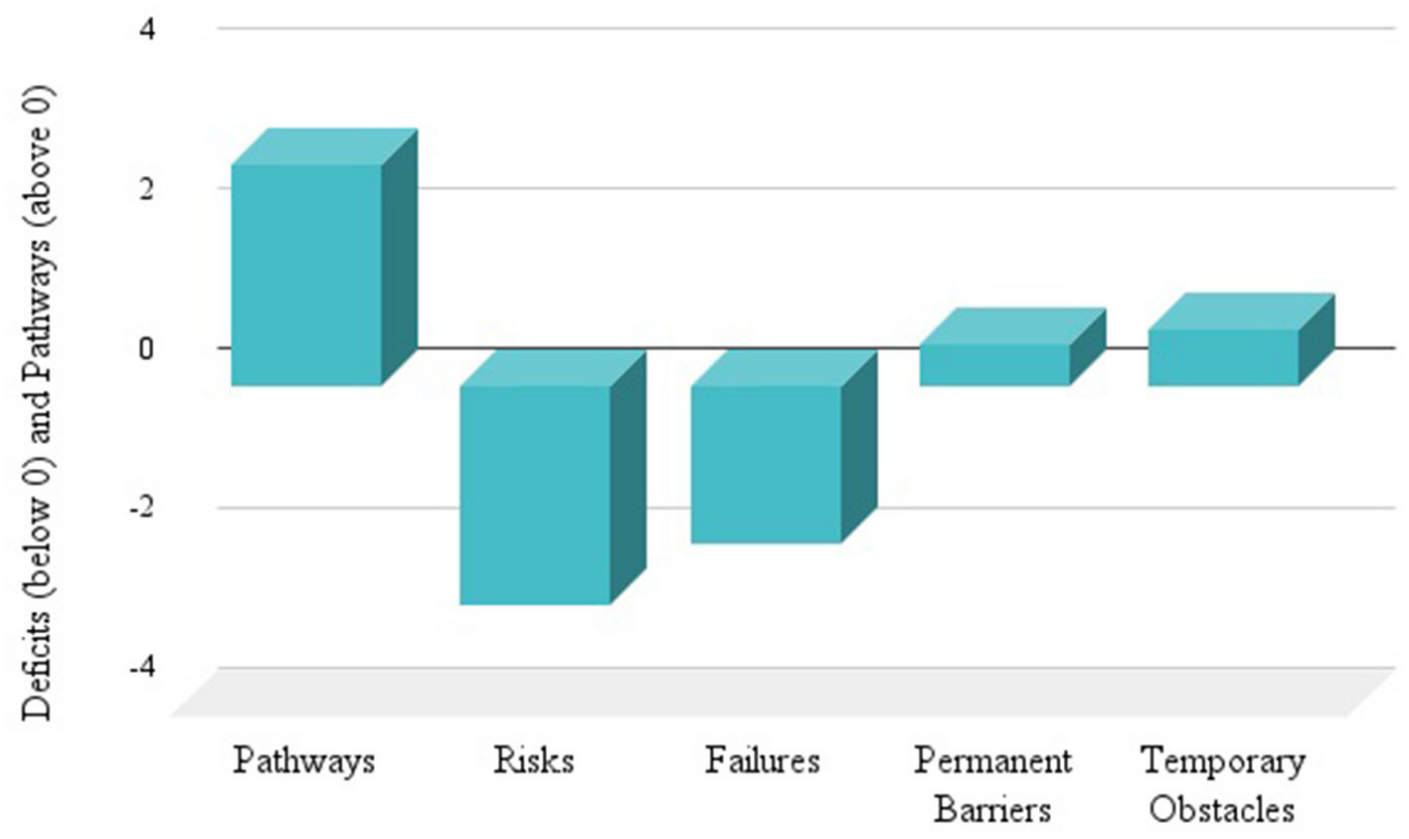
Comparison of Accessible Pathways and Features—shows the average pathway rating as a positive score and contrasts it to the average ratings of observed access risks and failures, permanent barriers, and temporary obstacles per community shows as deficits along the negative scale. Permanent barriers and temporary obstacles contribute to Access Failures. Access Risks and Failures reduce the Pathway Score.

We were also able to evaluate Access Risks and Failures associated with Permanent Barriers and Temporary Obstacles. Figure 3 portrays these features. Importantly, we observed 1.95 Access Risks and 1.40 Access Failures per 1,000 m of pathway. Many permanent obstacles appeared in the public right of way, labeled as Right of Way Obstructions.

#### Access and participation

We observed 561 people, an average of about 24 per community. The Rule of Proportional Participation (RPP) suggests that individuals who use mobility devices might be expected to be present in the same proportion as their prevalence in the population, or about 25 individuals. We observed 12 people using mobility devices, 48% of the RPP.

**Figure 4:**
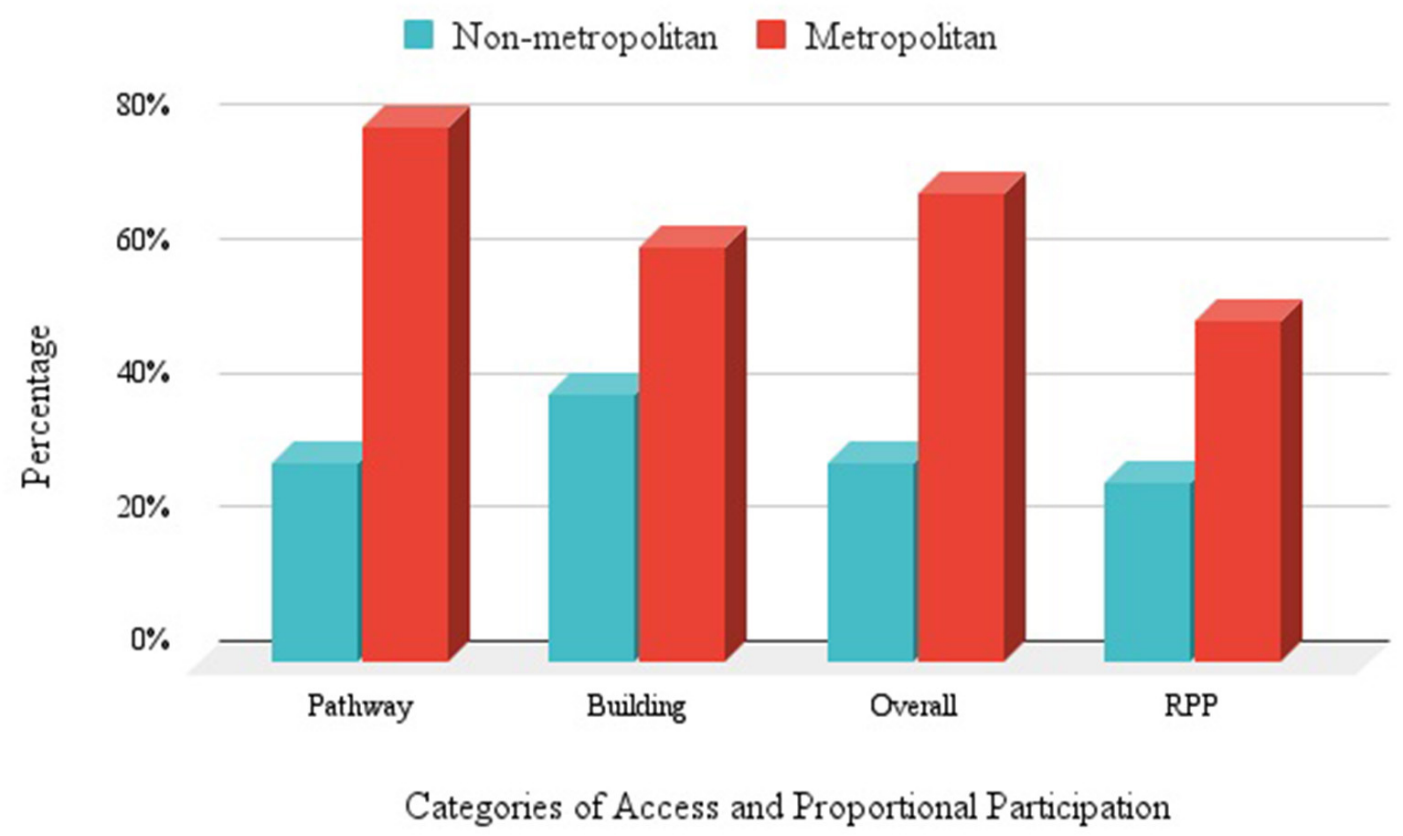
Rural Access Penalty and Rural Participation Penalty—shows non-metropolitan (blue) vs. metropolitan (red) access features (Pathways, Buildings, and Overall Scores) and participation as measured by the Rule of Proportional Participation (RPP). The metropolitan access and participation rates are consistently higher than non-metropolitan rates.

The Combined Access Score statistically predicted the proportion of people using personal mobility devices of all those observed (*r*_T_ = 0.277, 95% CI = [−0.026,0.58], *p* = 0.036). We noted consistent disparities between the levels of access and participation in non-metropolitan cities and those in metropolitan cities, which we labeled “Rural Access Penalty” (Figure 4). Importantly, participation averaged 27% of the expected rate under the Rule of Proportional Participation in non-metropolitan areas and 51% in metropolitan areas. The finding of lower rates of participation in less accessible rural areas supports both the commonsense argument and our hypothesis that participation in events at a place may be influenced by the accessibility of the place. However, the small number of towns located in non-metropolitan counties (26) and the low levels of observed presence of people with mobility impairments in those places (1) yielded no statistically significant results.

Similarly, we reasoned that participation might be influenced by the number of impediments encountered. We derived a new score, the Threat Access Ratio, by calculating the inverse of the proportion of inaccessible buildings (i.e., rated “0” in doorway or entryway) multiplied by the inverse of the sum of the Access Risks and Failures as a proportion of observed opportunities. We examined the correlation between the Threat Access Ratio and the proportion of people using personal mobility devices to all those observed (*r* = 0.23, *p* = < 0.14). While this derived measure is not significant in this sample, the sample is relatively small. A larger sample may show a relationship with this derived measure, and it would be worth considering it in the future.

### Discussion

This study demonstrated a relatively inexpensive and reliable method for evaluating the accessibility of communities and participation in them. While limited in the sample, the results suggest that, on average, even the most public of civic places are not universally accessible; with public pathways averaging 67% and buildings just 63% on our Combined Access Score. Nonetheless, these data provide empirical support for the assertion that the accessibility of a place influences the rate of presence of people who use mobility devices. Further, these results suggest that the burden of inaccessible places may fall disproportionately on rural residents of non-metropolitan counties. Indeed, while the overall Participation Score is just 52% of the RPP, rural residents participate at half that rate. Given the discrepancy in accessibility, this finding presents evidence to support the existence of a Rural Access Penalty.

Of course, these data and findings need to be interpreted with caution. First, our sample was limited in the number of communities observed and the places within a community observed. A larger, stratified sample of places would be helpful to create an accurate baseline. Similarly, the locations observed within each place were for convenience. Here, we chose the city hall as an anchor point to trace a path of 1,000 m. The selection of the 1,000 m was standard but arbitrary. Further, it treated all towns and cities, regardless of population or size, as the same. Other means for selecting areas for observation might be considered. Especially for larger places, more locations or a larger area might be sampled to develop a representation of the community. Community functions are often organized by location. Most cities and towns create zoning to do this or to shape it. Even within zones, there may be distinct groupings around functions that might need to be sampled. Larger samples would allow researchers to test hypotheses contained in this study and a wide range of additional ones adequately.^2^

## Study 2

Our experience in Study 1 suggested that the choice of community area to be assessed was inadequate and difficult to apply. The procedure oversampled places in small communities and under sampled features in larger communities. Moreover, the selection of areas in larger communities was arbitrary. Study 2 was designed to explore an alternative that involved assessing a community's central business district– a community's core area of public participation. Study 2 was conducted to support community action teams (CATs) working to advance community development opportunities.

### Sample

We worked with 22 towns and cities in one large, rural state including two communities on American Indians reservations. Communities were selected based on their participation in a state Healthy Communities' program (27–30). Towns and cities organized CATs to participate in the program and support the implementation of related community action plans (CAPs). CATs included community decision-makers and were supported to include representation of disability advocates and partner organizations on the CAT or in the implementation of CAPs (31–33). The towns and cities varied in size, with populations under 5,000 people (*n* = 7), between 5,000 and 10,000 people (*n* = 9), between 10,000 and 50,000 (*n* = 3); and over 50,000 people (*n* = 3) (Median population size = 6,681). The total population across all towns and cities was nearly 394,000 people.

### Procedures

#### Places observed

As an alternative to the methodology for selecting the area of a community to observe used in Study 1, we selected a standard unit area across communities and rated the accessibility of selected features of part or all of the area. Cities and towns are frequently organized around zones. For example, most communities in the United States include residential, commercial, industrial, and mixed zones. While a community may have several such zones, a community typically has one central business district (CBD) or downtown core. A community's CBD is its economic, cultural, governmental, and civic center. The core is characterized by multi-story buildings that primarily contain commercial, office, and retail land uses, as well as multiple surface parking lots and structures and institutional facilities. A limited number of residential structures are located in the CBD or downtown core, and those are typically multi-family. While the size and composition of a CBD vary from town to town, it is a recognizable unit. Moreover, the CBD is a focal point for participating in community life. As such, the accessibility of a CBD is central to the participation of people who experience disability. Also, the heathy community's program was promoting strategies and funding mechanisms targeting CBD policy, systems, and environmental changes, and we reasoned that CATs with accessibility assessments could support related community action plans that would have greater reach to people with disabilities. For these reasons, we chose to focus on assessing the accessibility of CBDs. The left panel of Supplementary Image 2 shows a CBD of a small town outlined with a yellow grid (Google Earth satellite view) and the right panel shows a GSV section of the CBD of another small town (GSV).

#### Measures and data collection

We used the same rating scale as we used in Study 1, with revisions to clarify scoring and the additional measures of sidewalk crossings (e.g., alleys and driveway that cross a sidewalk), street crossings (e.g., crosswalks), and railroad crossings, as well as counts of designated accessible parking spaces observed. We did not observe for temporary and permanent barriers. We calculated the same Access Scores for each of the features of each CBD. Access Scores included Pathway Access Scores overall and for curb cuts, sidewalk segments, sidewalk crossings, street crossings, and railroad crossings; Building Access Score overall and for building approaches and entries; Combined Access Score; and Designated ADA Parking Access Score.

#### Observational procedure

There is no universal list of downtown or CBD coordinates. Due to this situation, a general selection step was required for each community. Procedurally, an observer first entered the name of a city and the state into Map Quest (or Google equivalent) and selected the option to display the locations of all banks, libraries, post offices, pharmacies, drycleaners, museums, movie theaters, department stores, shopping centers and malls, florists, retail apparel stores, bookstores, office supply shops, parking garages, public transportation stations, and restaurants and bars. The image produced would suggest areas that may qualify as a CBD based on the density of businesses.

Next, the observer entered the name of the city and state into the Google Earth Search Bar. Once Google Earth presented the image of the city, the observer adjusted the elevation of the “eye altitude” to allow the entire city to be in view. Then, the observer scanned the geography for indicators of the CBD candidates. From above, these areas present images of groups of flat-top buildings that occupy relatively larger areas than the majority of structure in the city (i.e., residential structures). Typically, these areas have less visible vegetation (e.g., trees) and wider streets or roads. A downtown area can be distinguished from a commercial strip or industrial area by closer examination.

Additionally, the observer entered key terms in the search bar successively: city hall, downtown, and central business district. If any of these areas appeared in a candidate area and no other candidate area contained those terms, it was deemed as the CBD. If the search did not reveal any area as containing the search terms or if they were located in several different areas, a closer inspection of each candidate area was conducted to determine which area met the accepted definition of a CBD.

Finally, the observer established an observation grid. using natural boundaries (e.g., rivers, foothills) and constructed boundaries (e.g., streets, roads, and railroads) as guides to mark areas of transition from primary core activities from residential, mixed, and industrial activities. The grid was formed to maximize the inclusion of commercial, civic, entertainment, and governmental facilities but to minimize inclusion of residential areas, manufacturing, and industrial areas. In some instances, it is desirable to draw a sample from the grid. In this study, we made observations of the entire grid selected for each CBD. Once established, the grid was reviewed by a second researcher who could agree with the choices or modify them. Both agreements and modifications were monitored. All observers were trained to inter-rater reliability criterion to score environmental features as described above.

## Results

Overall, we evaluated 22 towns and cities in a large rural state. We observed ≈77,000 m (48 miles) of pathways through the observed CBDs, and scored 4,474 pathway features (1,547 curb ramps, 1,542 sidewalk segments, 822 sidewalk crossings, 545 street crossings), and 4,479 building features (2,258 approaches and 2,221 doorways). GSV images were newest for the two most populous communities (dated 0–2 months prior to our observations), whereas images for the rest of the communities were older (dated 25–89 months prior to the observations).

The Combined Access Scores across CBDs averaged 83.9%, including an average Pathway Access Score of 85.2% and an average Building Access Score of 83.0%. No community received a perfect score across these categories. More than half (52.3%) of the smaller communities with populations under 10,000 (*n* =16) had Combined Access Scores below the median (85.7%) while only a third (33.3%) of the communities with larger populations had Combined Access Scores below the median.

**Figure 5:**
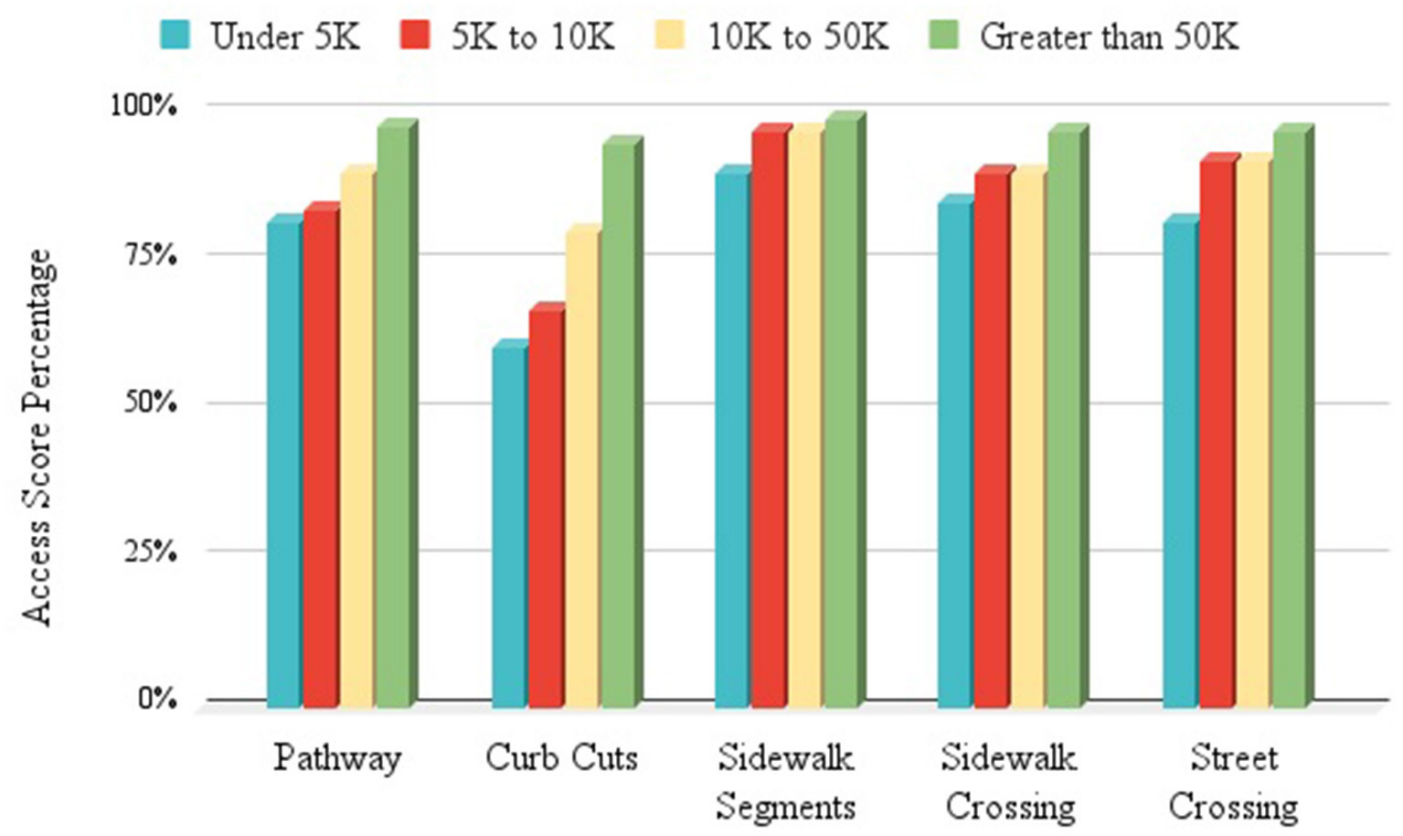
Access Scores by Community Population Groups—shows access feature scores (Pathway, Curb Cuts, Sidewalk Segments, Sidewalk Crossing, Street Crossing) by community population groups ranging from under 5,000 to >50,000 people. Communities with populations >50,000 score highest across all features.

Community population size was positively associated with all Access Scores, but this relationship was significant only between population size and curb cut (CC) scores (*r* = 0.42, *p* < 0.05) and between population size and the accessibility of designated parking spots (*r* = 0.46, *p* < 0.05). The positive relationship between community population size and overall Pathway Access Scores was on trend toward significance (*r* = 0.40, ns). Figure 5 presents mean Access Scores for Pathway features by four types of communities grouped by: populations under 5,000 people (*n* = 7); populations between 5,000 and 9,999 people (*n* =9); populations between 10,000 and 50,000 people (*n* = 3); and populations over 50,000 people (*n* = 3). Communities with populations over 10,000 had CBDs with higher average Pathway Access Scores, while the groups with populations under 5,000 had CBDs with the lowest average Pathway Access Scores for any feature. Mean Pathway Access Scores that decreased with the population sizes of community groups indicated support for a Rural Access Penalty. Curb cuts in CBDs in small, rural towns (under 5,000) had the lowest average Pathway Access Score of all average scores.

**Figure 6:**
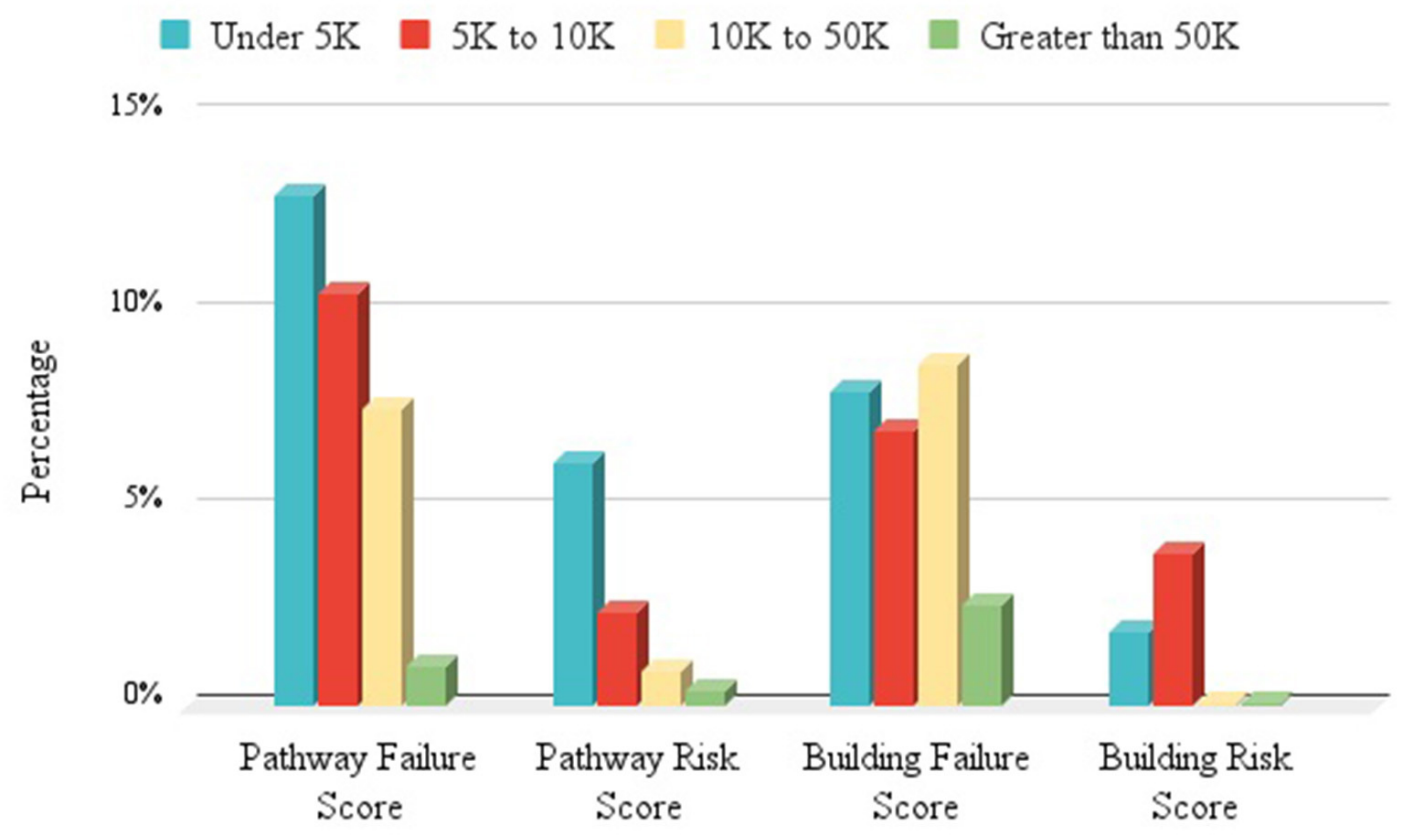
Failure and Risk Scores by Community Population Groups—shows pathway and building failure and risk scores by community groups ranging from under 5,000 to over 50,000 people. Failure and Risk Scores reflect the percentage of features that were scored a zero (failure) and the percentages scored a one (risk) of the total number of features scored. The Building Risk Scores for the two more populous groups of communities were <0.1%.

In addition to rating the accessibility, the number of pathway and building risks and failures were derived from observations. Failures reflect ratings of “0” for any feature; meaning that feature created an insurmountable obstacle to proceeding. Risks reflect a feature scored as “1,” a feature that exposed a person to a dangerous situation (e.g., divert into traffic), if they were to proceed. Figure 6 presents Pathway and Building Failure and Risk Scores by groups of communities ranging from under 5,000 to over 50,000 people. Failure and Risk Scores reflect the percentage of features that were scored a “0” (failure) and the percentages scored a “1” (risk) of the total number of features scored. The Building Risk Scores for the two more populous groups of communities were <0.1%.

Overall, 7.2% of the 4,474 features failed to provide an accessible pathway. Only 1.9% of pathway features presented a risky situation. The percentage of pathway features that failed exceeded 15% in six of the communities, all with populations under 10,000 people. One rural and one urban community presented a completely accessible (no Access Failures) CBD infrastructure.

Observers noted seventy-one designated parking spaces, receiving an average access rating of 1.96. Five of the spaces were observed to be in use. No designated spaces were observed in five communities.

GSV images showed people in only three CBDs (two rural and one urban community). Observers noted 56 individuals in the areas observed. Of those, 3.6% used a mobility device. The Rule of Proportional Participation suggests that 4.5% of those observed should be expected to use a mobility device or 2.52 individuals.

## Discussion

The observation system focusing on a city's CBD performed well. Failure and Risk can be attributed, in part, to the location of the CBD. For example, one city's CBD is located on a major highway. When the highway was refurbished, the State and City arranged a complete rehabilitation of the sidewalks in the CBD. In addition to creating accessible pathways, the reconstruction was done in a way to bring the sidewalks very close to the level of many old buildings. As such, this improved the accessibility of both the public pathways and the privately-owned buildings. Similar designs and arrangements have produced significant increases in accessibility in several small towns in the State.

A future direction is use of this method to evaluate change over time and implications of community action planning and related policy, systems, and environmental changes (34–37). For example, between 2013 and 2018, the communities in Study 2 participated in annual healthy community workshops, with half of these communities choosing to participate in multiple workshops. After the workshops, 10 communities developed and passed complete streets policies. Further, six communities created and implemented motorized transportation plans, and four designed non-motorized transportation plans. Other community plans were also developed, including downtown master plans (*n* = 5), growth policies (*n* = 3), and wayfinding plans (*n* = 2). Six communities generated both a complete streets policy and another community policy or plan, such as a transportation plan or wayfinding plan. As of 2018, there were 24 active transportation plans and policies in communities statewide, including complete streets policies and master plans (motorized and non-motorized transportation plans). Additionally, two of the communities were receiving technical assistance on the ADA through the U.S. Department of Justice's Project Civic Access, as a separate activity from the healthy communities program. Community leaders could use the current method to evaluate the impact of their community action planning on changes over time using available GSV data. e.g., within an inclusive, interdisciplinary audit workshop (38, 39). Indeed, the full version of Google Maps affords access to old street-level imagery from the GSV archives to support reviews of community change over time. These archives also would allow a closer study of imagery updates in rural and urban places.

Finally, Healthy People 2030 (40) has a *Community* goal to “Promote health and safety in community settings” that currently organizes 20 HP2030 objectives to achieve this goal. The current method could support partners working on these objectives to plan for increased accessibility of health promotion opportunities in community settings across their efforts. For example, the HP2030 *People with Disabilities* workgroup could provide leadership on how to integrate this method and similar tools into HP2030. Additionally, this workgroup could support increased use of environmental data across HP2030. Organizing for environmental interventions is necessary to eliminating health disparities experienced by people with disabilities within an ecological framework and the bio-psycho-social model of disability.

## Conclusion

These two studies demonstrate a method for using GE and GSV to conduct distance observations of accessibility of rural and urban communities. Together, they suggest that disparities exist between rural and metropolitan cities, such that rural areas have poorer accessibility ratings, leading to decreased community participation. This disparity is termed as a “Rural Access Penalty.” Continued monitoring and use of such data to plan and evaluate infrastructure investment of community accessibility, particularly in rural areas, is critical for community members' health and quality of life. Advocates may also find results from more cities useful.

These studies demonstrate the usefulness of GSV in measuring features of accessibility. This approach also produced operational definitions for several potentially useful concepts. Several of those either emerged from observation (e.g., Access Islands) or were derived by combining measures (e.g., Threat Access Ratio). Others were identified but are not reported here. This work suggests additional benefits to this scientific approach to monitoring accessibility and representing these concepts and related design considerations in planning for infrastructure development

It seems quite feasible that future research could develop an algorithm for noting and scoring accessibility features of images that would permit for computerized scoring of GSV images (41). This would increase the feasibility of nationwide accessibility assessments. An explicit partnership would be needed to replicate, routinely repeat, and expand this method systematically.

GSV has its limitations and drawbacks. First, GSV is limited to those aspects of the arranged environment that are detectable by visual inspection, and to measures derived from those observations. The level of observation limits analysis to the information captured by a car-mounted camera as it drives a route through a place. The time of day, the day of the week, and the season of the year are determined and may not reflect the needs of accessibility evaluations. Nonetheless, as in wildlife biology, multiple levels of analysis are used to assess the habitat of a population, the population in interaction with the habitat, and the behavior of individuals. Indeed, such an ecological model could be applied to organize the existing literature and guide additional research in the study of disability. As with the natural sciences, understanding at those levels could be integrated into public policy and practice, and serve as the basis of the development of the science.

Second, GSV has been criticized over concerns for privacy (42). In one case, Google was fined for intrusions in multiple states and countries and has since apologized for these actions. Further, Google has taken measures to protect privacy in GSV images, including blurring out the faces of individuals present and blurring license plates. The use of GSV imaging is legal, and studies using these images should engage in ethical measures to protect confidentiality by following ethnographic, observation, and participatory action research guidelines. When used ethically, GSV can benefit communities.

Third, GSV data represent static, one-time, cross-sectional observations. Participation is a dynamic process. As such, it requires a dynamic measure. Importantly, the environment is also in flux, and it too calls for measures across time. For example, the features of an arranged environment can interact. A curb ramp installed today may sit astride a water main buried directly below. A new building, a change in policy or practice, or simple routine maintenance may lead to the installation of a fire hydrant in the middle of the curb ramp tomorrow. Similarly, sidewalks deteriorate over time and their characteristics change. If viewed on typical periods familiar to rehabilitation researchers focusing on individual behavior, the arranged environment can be treated as relatively stable. Viewed from a perspective of natural resource management or civil engineering, the timeframe of the succession of flora or the lifetime of a bridge can be a 100 years.

This study, along with the research of others, demonstrates the value of scientific measurements of environment and participation. Society invests a great deal in scientific approaches to monitoring and managing the natural and constructed environment. For example, the U.S. Forest Service (43), the Fish and Wildlife Service (44), and the U.S Geological Service (45) collect a wide range of data from space and on the ground that permit analysts to assess the health of entire forests, including the moisture content of soils, the spread of diverse vegetation throughout habitats, as well as the populations and individuals that inhabit them (46). Similarly, the American Society for Civil Engineering (47) monitors the condition of America's infrastructure, including our bridges, dams, drinking water plants, levees, public parks and recreation facilities, schools, and transit systems. Findings from such programs are integrated into policy development, used for modifying program practices, and serve as a basis for improving science. Yet, there is no such program assessing the accessibility of our communities or participation in them. This lack of information hampers policy development and program practice.

The emerging science of the 21st century will be a science of the environment (48, 49). This movement has generally focused on models in which exposure to an environmental variable over time produces a disease response; however, this model can also be used to understand the impact of environmental risk and protective factors for community participation. Programs intended to evaluate the impacts of disability policies and programs have been scattered across Federal agencies, private organizations, and independent researchers with little integration of information (50–54). New technology provides a means for enhancing the scientific understanding of the effects of environmental factors on participation in community life. Future research may also use this technology to provide a foundation for assessing additional factors of environments (e.g., cognitive, sensory) and other areas (e.g., web sites and voting places). These data could be coordinated and consolidated by one central program with a responsibility for integrating it into public policy and practice, and into the development of the science. As such, there is both a need for and possibility of organizing a national laboratory on the environment and participation.

## Data availability statement

The original contributions presented in the study are included in the article/Supplementary materials, further inquiries can be directed to the corresponding author.

## Author contributions

Data collection and analysis for the project was done by TS and MT. The writing was done by TS, MT, and EH. Critical review and suggested edits were completed by all authors. All authors contributed to the article and approved the submitted version.

## Funding

This research is supported by a grant from the National Institute on Disability and Rehabilitation research (NIDRR), U.S. Department of Education, #H133B080023. This project received additional support from the Research and Training Center on disability in Rural Communities (RTC:Rural) under a grant from the National Institute on Disability, Independent Living, and Rehabilitation Research (NIDILRR; Grant number 90RTCP0002). NIDILRR is a Center within the Administration for Community Living (ACL), Department of Health and Human Services (HHS). This project also had support from cooperative agreements between the Centers for Disease Control and Prevention (CDC), U.S. HHS and the Montana DPHHS (DD16-1603; DD21-2103). EH receives/received support from Montana INBRE97an Institutional Development Award from the National Institute of General Medical Sciences of the National Institutes of Health under Award Number P20GM103474. The work does not necessarily represent the policy of NIDILRR, ACL, CDC, NIH or HHS and one should not assume endorsement by the federal government.

## Conflict of interest

The authors declare that the research was conducted in the absence of any commercial or financial relationships that could be construed as a potential conflict of interest.

## Publisher's note

All claims expressed in this article are solely those of the authors and do not necessarily represent those of their affiliated organizations, or those of the publisher, the editors and the reviewers. Any product that may be evaluated in this article, or claim that may be made by its manufacturer, is not guaranteed or endorsed by the publisher.

## Footnotes

^1^A complete set of definitions and training materials may be obtained from the second author.

^2^Based on these preliminary studies, we estimated that a sample of 2,219 communities in each population range (242 for communities over 49,999 people, 338 for communities between 10,000 and 49,999 people, and 1,639 for communities of <10,000 people) would provide a statistically valid representation of the 36,000 communities in the United States, sovereign American Indian reservations, and territories.
