# Supplementary figures and images for: Exploring environmental measures in disability: Using Google Earth and Street View to conduct remote assessments of access and participation in urban and rural communities
Source: Front Rehabil Sci. 2022 Aug 5;3:879193. doi: 10.3389/fresc.2022.879193 (PMC9397703; doi:10.3389/fresc.2022.879193)

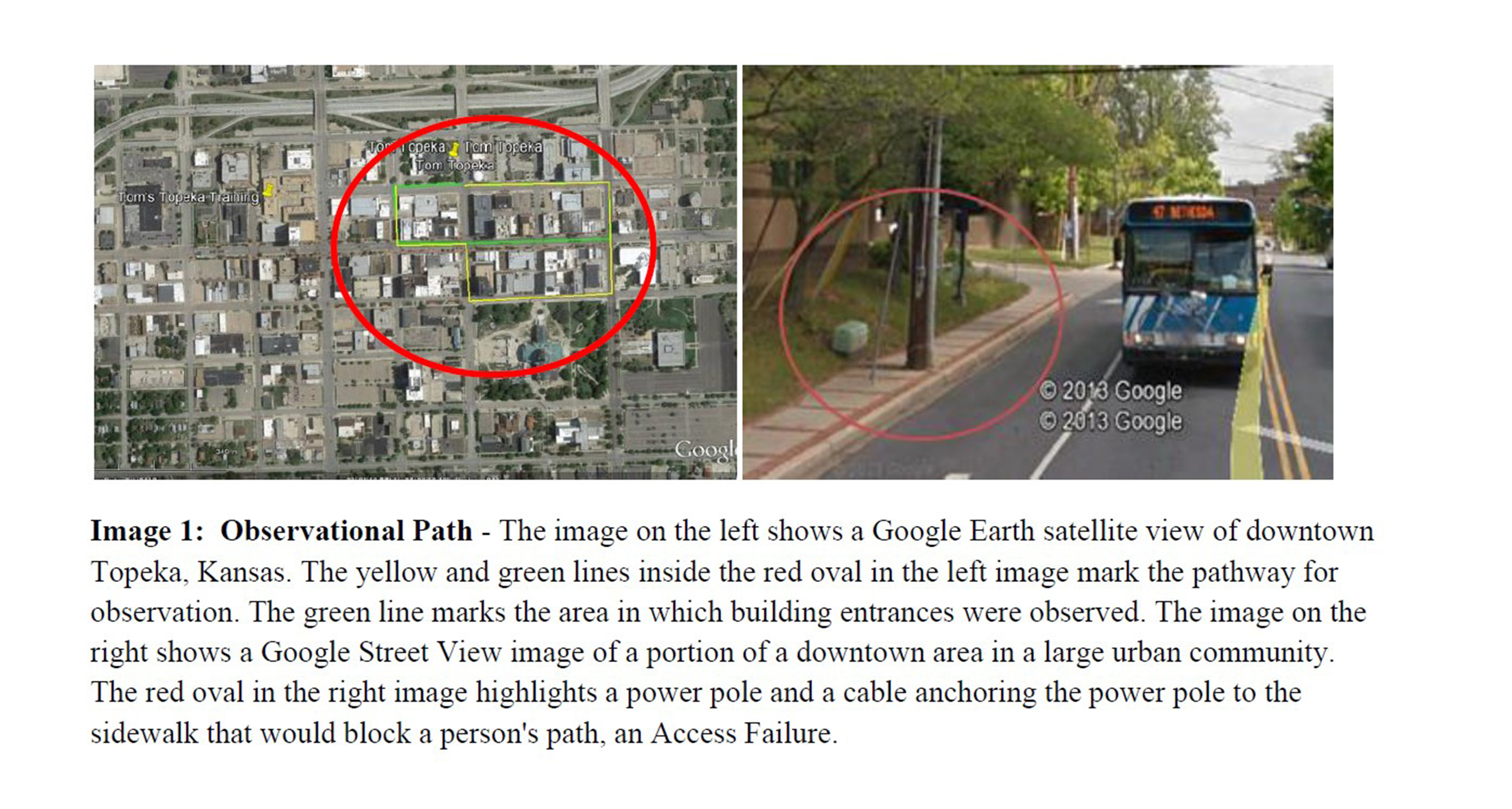

Supplement: Supplementary file 1 [file Image_1.JPEG]

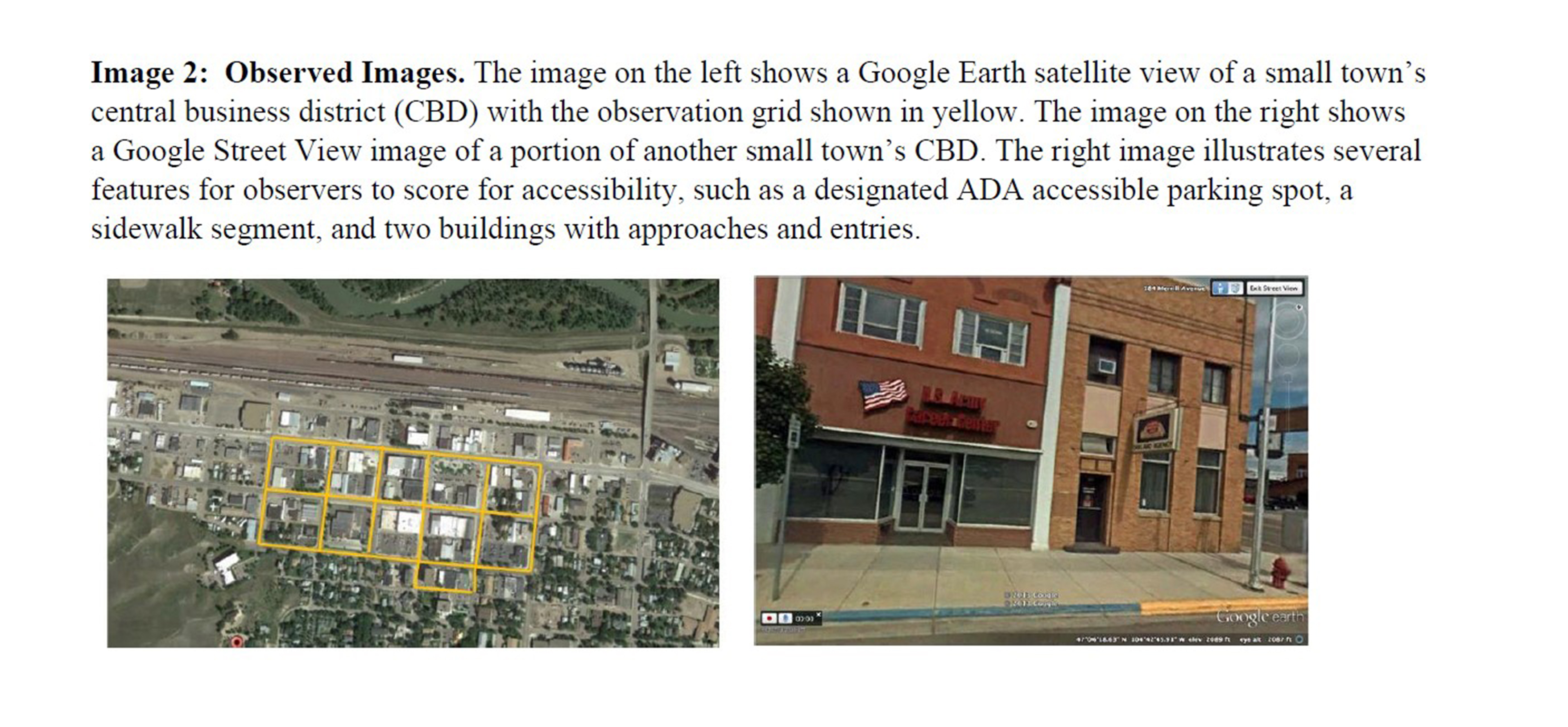

Supplement: Supplementary file 2 [file Image_2.JPEG]
